# Identification of Key Genes Involved in Resistance to Early Stage of BmNPV Infection in Silkworms

**DOI:** 10.3390/v14112405

**Published:** 2022-10-29

**Authors:** Linyuan Yu, Yeqing Cao, Sicheng Ge, Anying Xu, Heying Qian, Gang Li

**Affiliations:** 1 The Sericultural Research Institute, Jiangsu University of Science and Technology, Zhenjiang 212018, China; 2 Key Laboratory of Silkworm and Mulberry Genetic Improvement of Agriculture and Rural Affairs, Zhenjiang 212018, China

**Keywords:** *Bombyx mori*, BmNPV, infection, RNA-seq, host–virus interaction

## Abstract

*Bombyx mori* nucleopolyhedrovirus (BmNPV) is one of the most serious pathogens restricting the sustainable development of the sericulture industry. Currently, there is no effective treatment for BmNPV infection in silkworms, and the mechanism underlying BmNPV resistance in silkworms is also not clear. In this study, comparative transcriptome analyses were carried out in midguts of two silkworm varieties, namely BaiyuN, which is a resistance variety, and Baiyu, which is a susceptible variety, at five different time points (i.e., 0, 1, 3, 6, and 9 h) post-BmNPV infection to detect the early-stage transcriptional changes in these silkworms. In total, 1911 and 1577 differentially expressed genes (DEGs) were identified in the Baiyu and BaiyuN varieties, respectively, involving a total of 48 metabolic pathways. Of these pathways, eight were shared by the Baiyu and BaiyuN varieties in response to BmNPV infection. Notably, four genes (i.e., BGIBMGA08815, BGIBMGA003935, BGIBMGA003571, BGIBMGA010059) were upregulated in the Baiyu variety while downregulated in the BaiyuN variety. The inhibited expression of these four genes in the resistant variety highlighted their potential roles in the resistance of early-stage viral replication. Thus, our study provided a new avenue for the further study of the mechanism underlying BmNPV infection in silkworms and the potential treatment of BmNPV infection.

## 1. Introduction

The silkworm, *Bombyx mori*, is an economically important insect in sericulture. With the publication of the draft and fine map of the *B. mori* genome, the silkworm has become an important model organism for the study of Lepidoptera. Viral diseases can cause severe threats to the growth and development of plants and animals [1,2]. *B. mori* nucleopolyhedrovirus (BmNPV) infection is a major threat to the sericulture industry and can cause serious economic losses [3,4,5]. Some silkworm varieties have developed excellent resistance to BmNPV infection. Our laboratory has also successfully cultivated “Huakang” series varieties that are highly resistant to BmNPV infection through hybridization. During recent decades, extensive studies have been carried out that enriched our understanding of the molecular mechanisms underlying silkworm resistance to BmNPV infection. However, the molecular mechanisms have not been fully elucidated [6,7,8].

As sequencing technology develops, a number of genes (e.g., amino acid transporter, serine protease, 26S proteasome, and heat shock encoding proteins) related to disease resistance have been identified from resistant varieties [9,10,11]. For instance, E3 ubiquitin ligase RNF128 is shown to play an important role in innate antiviral immunity [12]. RNA-sequencing (RNA-seq) is an effective and powerful tool for transcriptome analysis [13]. For example, using transcriptome analysis, a subset of genes responsive to biotic or abiotic stress were identified that revealed novel defense mechanisms in some species [4,9,14].

Silkworms at different stages of viral infection may have different transcriptional profiles, but host factors essential for viral replication or resistance may be identical at these stages. The midgut of silkworm serves as the first physiological barrier to virus infection and plays an important role in virus replication and host defense [15,16]. Previous studies reported that some specific genes in the midgut of silkworms play crucial roles in virus–host interaction [16,17]. Thus, identification and characterization of differentially regulated genes in larval midguts at different stages post-virus infection may help us to identify essential common host factors for virus replication and host resistance. To date, no effective strategies have been available to control BmNPV infection in silkworms. We hypothesized that silkworm defense against virus infection is a rapid process in the early infection stage. In order to gain a better understanding of the mechanisms underlying resistance to BmNPV infection in silkworms, we comparatively analyzed the transcriptional profiles of larval midguts of susceptible and resistant silkworm varieties at different time points post-BmNPV infection. As a result, many differentially expressed genes (DEGs) were identified. Of them, seven genes showed the same trends (either downregulated or upregulated) in the susceptible silkworm variety at different stages of BmNPV infection, indicating that these genes might be relevant to the BmNPV infection process. Furthermore, several new transcripts were predicted. In summary, our study provides a valuable resource for further identification of genes involved in the resistance of silkworms to BmNPV infection and offers new insights into the molecular mechanism underlying *Bombyx mori*–BmNPV interactions.

## 2. Materials and Methods

### 2.1. Preparation of BmNPV and Silkworm Midgut Samples

BmNPV was maintained in our laboratory. The BmNPV virus was propagated in BmN cells, which were maintained at 27 °C in TC-100 insect medium (Gibco, New York, NY, USA) that was supplemented with 10% (*v*/*v*) fetal bovine serum (Gibco). The titration of virus and other routine manipulations were performed according to standard protocols. One milliliter of BmNPV viral suspension was added to 100 μL of kanamycin (50 mg/mL) and 100 μL of gentamicin (7 mg/mL). The silkworm *B. mori* varieties, BaiyuN (an infection resistant variety) and Baiyu (an infection susceptible variety), were maintained in our laboratory and reared using fresh mulberry leaves under laboratory conditions at 27 ± 1 °C with a 70–85% relative humidity and photoperiodic lighting (16h of light/8h of dark). The BmNPV infection of silkworms was performed orally according to the method reported by Li et al. [4]. Briefly, each of the 50 newly molted silkworm larvae of the 5th instar from each variety was treated orally with 7 μL of BmNPV suspension (1.0 × 10^9^ polyhedra/mL). For each variety, the larvae were then divided into five groups randomly. Specifically, the susceptible variety Baiyu (B) groups were named B-0, B-1h, B-3h, B-6h, and B-9h, and the resistance variety BaiyN (BN) groups were named BN-0, BN-1h, BN-3h, BN-6h, and BN-9h, respectively. The midgut tissues were harvested at 0, 1, 3, 6, and 9 h post-infection on ice (samples were collected in triplicate for each time point) and were flash-frozen in liquid nitrogen. The collected tissues were kept at −70 °C until further use.

### 2.2. RNA Extraction and RNA-Seq

Total RNA was extracted from midgut tissues with TRIzol LS (Invitrogen, Los Angeles, CA, USA) according to the manufacturer’s instructions. The total RNA was treated with RNase-free DNase I in order to remove genomic DNA contamination, as previously described [4,18]. The purity and quality of the RNA were determined using a NanoDrop ND-1000 Spectrophotometer (NanoDrop, Wilmington, DE, USA), with an OD260/280 value of 1.9–2.0 indicating good quality. Concentrations were quantified using a Qubit 2.0 Fluorometer (Invitrogen Corporation, Carlsbad, CA, USA) following the manufacturer’s instructions. The integrity of the RNA was confirmed by using an Agilent 2100 Bioanalyzer (Agilent, Santa Clara, CA, USA) with RNA integrity number (RIN) values over 8.0 indicating good integrity. The clustering of the index-coded samples was performed on a cBot Cluster Generation System using TruSeq PE Cluster Kit v4-cBot-HS (Illumia) according to the manufacturer’s instructions. After cluster generation, the library preparations were sequenced on an Illumina HiSeq 2500 platform (Illumina Lnc., San Diego, CA, USA), and paired-end reads were generated. Three biological replicates were used to minimize sample differences.

### 2.3. RNA-Seq Data Analysis

Raw data (raw reads) of fastq format were firstly processed through in-house Perl scripts. In this step, clean data (clean reads) were obtained by removing reads containing adapters, reads containing ploy-N, and low-quality reads from raw data. At the same time, Q20, Q30, GC-content, and sequence duplication level of the clean data were calculated. All the downstream analyses were based on clean data with high quality. In order to obtain clean and high-quality reads for sequence assembly, the raw data were processed to remove adapter sequences and low-quality reads (more than 50% of low-quality bases with Q-value ≤ 5 in a single read). Clean data were mapped to the silkworm reference genome downloaded from the SilkDB database (http://silkworm.swu.edu.cn/silkdb/) using the TopHat2 software [19]. The gene expression levels were quantified with fragments per kilobase of transcript sequence per millions base pairs sequenced (FPKM) [20]. DEG analysis was performed using Cuffdiff. For each comparison group, DEGs were identified with an absolute log2 ratio ≥ 1, the threshold fold change ≥ 2, *p*-value < 0.05, and false discovery rate (FDR) < 0.01.

### 2.4. Functional Annotation

Moreover, Gene Ontology (GO) and Kyoto Encyclopedia of Genes and Genomes (KEGG) were analyzed for significantly enriched DEGs using hypergeometric distribution for each KEGG pathway and GO term [21]. Fragments per kilobase of transcript per million fragment mapping (FPKM) was used to represent gene expression, and EBSeq was used for differential analysis. DEG screening criteria were as follows: |log2 (fold change)| > 2, *p* < 0.05 and FRD < 0.01. The DEGs were compared by COG, GO, and KEGG databases, and their functional annotations were analyzed [22,23].

### 2.5. Verification of DEGs by qRT-PCR

According to KEGG-enriched pathways, 10 DEGs were selected for validation by qRT-PCR. The primers were designed by Primer Premier 6.0 software and are listed in Table S1. RNA samples were from the same batch of transcriptome sequencing samples. The total RNA was extracted using the EASYspin Plus tissue/cell RNA rapid extraction kit (Beijing Aidelai Biotechnology Co., Ltd., Beijing, China), and reverse transcribed into cDNA. qRT-PCR was performed using the ChamQ Universal SYRB qPCR Master Mix kit (Nanjing Novizan Biotechnology Co., Ltd., Nanjing, China) according to the manufacturer’s instruction on a LightCycler^®^ 96System (Roche, Basel, Switzerland). The thermal cycling was as follows: initial denaturation at 95 °C for 5 min, followed by denaturation at 95 °C for 5 s and 60 °C for 30 s, for a total of 40 cycles. *BmActin-3* was used as a reference gene (U49854). The relative expression of *BmActin-3* was calculated using the 2^−ΔΔCT^ method [24].

### 2.6. Data Statistics

GraphPad Prism8 software was used for the independent sample *t*-test. All results were presented as mean ± standard error; *p* < 0.05 indicated a significant difference.

## 3. Results

### 3.1. Resistance of the Two Silkworm Varieties to BmNPV Infection

To verify the difference in resistance between the two varieties, the median lethal concentration (LC50) was used to evaluate the resistance level of silkworms to BmNPV infection. As a result, the median LC50 value in the BN group was 10,000-fold higher than that in the B group (Table 1) [25].

### 3.2. Overview of the RNA-Seq Data

Transcriptome profiling is an efficient technology that can compare gene expression differences in an unbiased manner. In our study, we aimed to use this technology to identify critical genes that relate to the response of susceptible (B) and resistant (BN) silkworm varieties to BmNPV infection at different time points post-infection. A total of 852,364,744 raw reads were obtained from 30 libraries, with the total read higher than 28,000,000 in each library (Table S2). After removing the adaptors and low-quality reads, 826,377,314 clean reads (96.95% of total reads) were left (Table 2). The ratio of reads with a Phred score ≥Q30 was higher than 94.55% for each library, the GC content of each sample was around 45%, and more than 77.41% of clean reads were mapped to the silkworm genome (Table 2), indicating good quality of the sequencing data, which could be used for further downstream analysis. Raw sequencing reads of all samples reported in this study have been deposited into the NCBI Sequence Read Archive from SRR20959739-SRR20959768 under the Bioproject PRJNA867118.

### 3.3. Overall Analyses of DEGs at Different Time Points Post-BmNPV Infection

**Figure 1:**
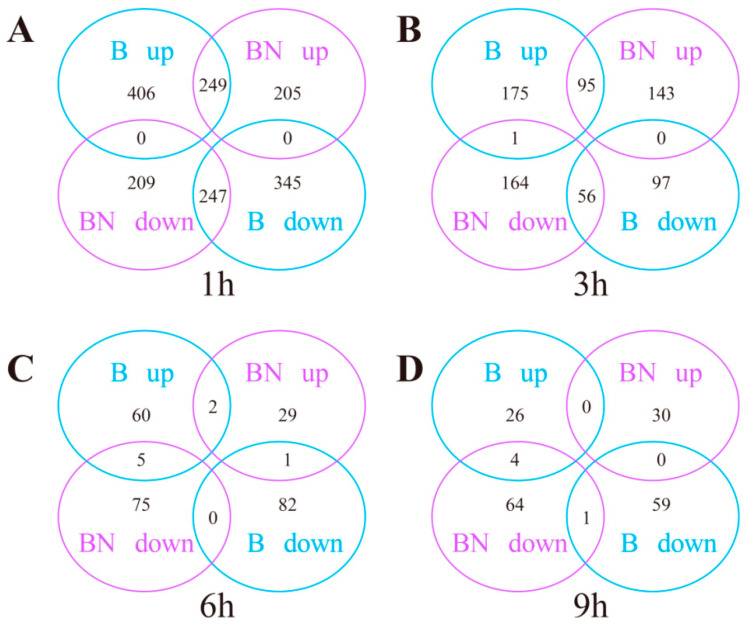
Venn diagrams showing DEGs in different comparison groups. The expression levels of B-1h, B-3h, B-6h, and B-9h, were compared to those of B-0h, while the expression levels of BN-1h, BN-3h, BN-6h, and BN-9h were compared to those of BN-0h, respectively. The up- and downregulated DEGs were identified by fold changes higher than 2.0 or lower than 0.5. (**A**) DEGs identified by comparing expression levels of B-1h and BN-1h. (**B**) DEGs identified by comparing expression levels of B-3h and BN-3h. (**C**) DEGs identified by comparing expression levels of B-6h and BN-6h. (**D**) DEGs identified by comparing expression levels of B-9h and BN-9h.

By comparison with the control groups (B-0 or BN-0), DEGs in the midgut tissues at 1, 3, 6, and 9 h in both varieties after infection were identified (Figure 1). A total of 2157 DEGs were identified in eight groups (B-1h vs. B-0, B-3h vs. B-0, B-6h vs. B-0, B-9h vs. B-0, BN-1h vs. BN-0, BN-3h vs. BN-0, BN-6h vs. BN-0, and BN-9h vs. BN-0), which are summarized in Tables S2–S4. Some of these DEGs were unique for certain groups, while others were shared in different groups (Figure 1). Thus, our results provide a comprehensive view of transcriptional changes in response to BmNPV infection in the two varieties. Furthermore, 6814 new transcripts were predicted from our sequencing data (Table S5).

**Figure 2:**
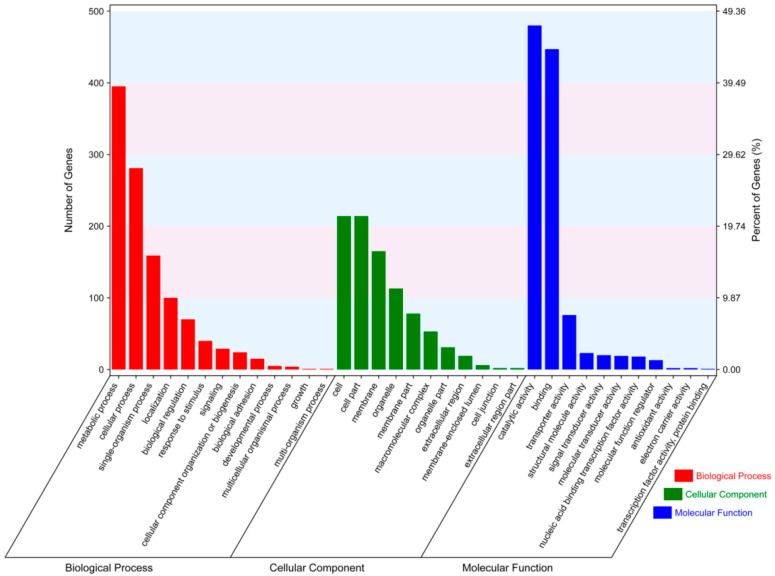
GO term enrichment analysis of the DEGs, classified based on BP, CC, and MF.

Gene ontology (GO) analyses were performed for the DEGs in the B-1h, B-3h, B-6h, B-9h, BN-1h, BN-3h, BN-6h, and BN-9h groups to identify enriched pathways in the cellular component (CC), molecular function (MF), and biological process (BP) subcategories. GO analyses showed that 935 DEGs were significantly enriched in four GO terms in the BP subcategory. Of these 935 DEGs, 35.1% were involved in the metabolic process. Regarding the CC subcategory, a total of 214 DEGs were enriched. Regarding the MF category, 940 DEGs were enriched in the catalytic activity (Table S2; Figure 2).

### 3.4. Transcriptome Changes at Different Time Points Post-BmNPV Infection

#### 3.4.1. Transcriptome Changes at Different Time Points in the Susceptible (B) Variety

**Figure 3:**
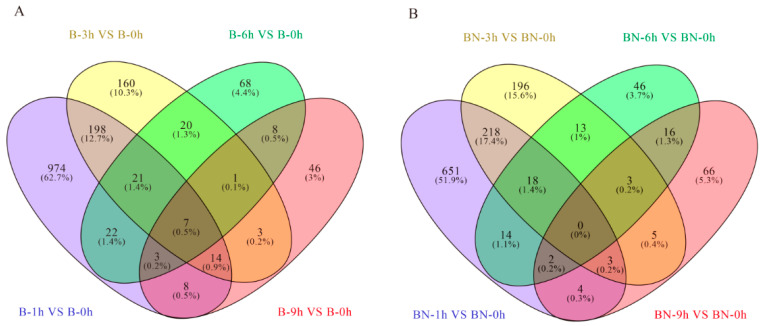
Venn diagrams showing the numbers of DEGs related to BmNPV infection in different resistant varieties. (**A**) Venn diagrams displaying the numbers of specific and shared DEGs in the B variety. (**B**) Venn diagrams displaying the numbers of specific and shared of DEGs in the BN variety.

Different transcriptome patterns were revealed when comparing transcriptome profiles of the B-1h, B-3h, B-6h, and B-9h groups with that of the B-0 group. In total, 1911 DEGs were identified in the B-1h, B-3h, B-6h, and B-9h groups (Figure 3A). More specifically, 655, 271, 67, and 30 DEGs were upregulated, while 592, 152, 83, and 60 DEGs were downregulated in the B-1h, B-3h, B-6h, and B-9h groups, respectively (Table S3). Notably, half of these upregulated DEGs were in the B-6h and B-9h groups (Table S3, Figure S1). Interestingly, we also noticed that the number of DEGs decreased with the extension of infection time (Table S3), indicating these DEGs might play an important role in defending BmNPV infections.

To further elucidate which DEGs have a potential role in the antiviral response, a Venn diagram was constructed (Figure 3A). As shown in Figure 3A, there are 974, 160, 68, and 46 DEGs in the B-1h, B-3h, B-6h, and B-9h groups, respectively (Figure 3A), indicating that the number of DEGs decreases as the BmNPV infection continues. Seven DEGs were upregulated at all the time points in the B variety (Figure 3A), implying that these seven genes may be involved in early compatible and incompatible interactions between B silkworms and BmNPV.

#### 3.4.2. Transcriptome Changes at Different Time Points in the Resistant (BN) Variety

Similarly, different transcriptome patterns were identified when comparing transcriptome profiles of BN-1h, BN-3h, BN-6h, and BN-9h groups with that of the BN-0 group. A total of 1577 DEGs were identified in the BN-1h, BN-3h, BN-6h, and BN-9h groups (Figure 3B). Similar to what has been observed in the B variety, more than half of the upregulated DEGs were in the BN-1h and BN-3h groups, while more than half of the downregulated DEGs were in the B-6h and B-9h groups (Table S3, Figure S1). Similarly, with the increase in infection time, the number of DEGs decreased (Table S3). Venn diagrams showed that 651, 196, 46, and 66 DEGs were in the BN-1h, BN-3h, BN-6h, and BN-9h groups, respectively (Figure 3B). Nonetheless, none of these DEGs showed the same change patterns at all the time points (Figure 3B), implying that the infection response in the BN variety is more complicated than that in the B variety.

### 3.5. Transcriptome Differences between the Susceptible (B) and Resistant (BN) Varieties

To explore the transcriptome differences between the B and BN varieties in response to BmNPV infection, the transcriptome profiles of the B and BN varieties at different time points were also compared. In the B-1h and BN-1h groups, there were 496 shared DEGs, with 249 genes being upregulated and 247 genes being downregulated in both groups (Figure 1). In the B-3h and BN-3h groups, there were 152 shared DEGs, with 95 genes being upregulated and 56 genes being downregulated in both groups, and one gene (i.e., a putative lipase (BGIBMGA009162) showing opposite changes in two groups (Figure 1). In the B-6h and BN-6h groups, there were only eight shared DEGs, with two genes being upregulated in both groups, five genes being upregulated in the B-6h group while downregulated in the BN-6h group, and one gene being downregulated in the B-6h group while upregulated in the BN-6h group (Figure 1). In the B-9h and BN-9h groups, there were only five shared DEGs, with one gene being downregulated in both groups and four genes being upregulated in the B-9h group while downregulated in the BN-9h group (Figure 1). It is worth noting that there were 205, 143, 29, and 30 upregulated DEGs in the BN-1h, BN-3h, BN-6h, and BN-9h groups compared with the B-1h, B-3h, B-6h, and B-9h groups, respectively (Figure 1), indicating that BmNPV infection can induce different transcriptional changes in the B and BN varieties.

### 3.6. KEGG Pathway Analysis of DEGs at Different Time Points

**Figure 4:**
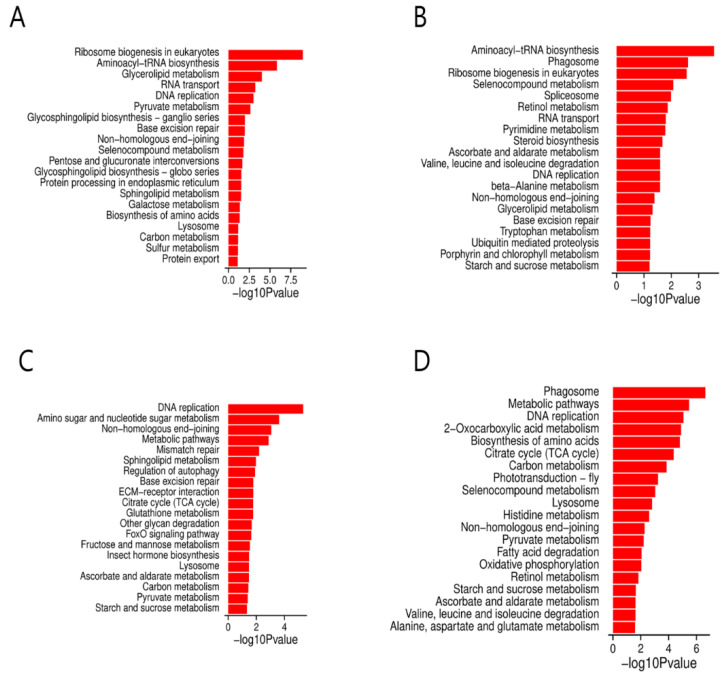
KEGG pathway enrichment analysis of the DEGs. (**A**) KEGG pathway enrichment analysis of the DEGs in B-1h. (**B**) KEGG pathway enrichment analysis of the DEGs in BN-1h. (**C**) KEGG pathway enrichment analysis of the DEGs in B-3h. (**D**) KEGG pathway enrichment analysis of the DEGs in BN-3h.

**Figure 5:**
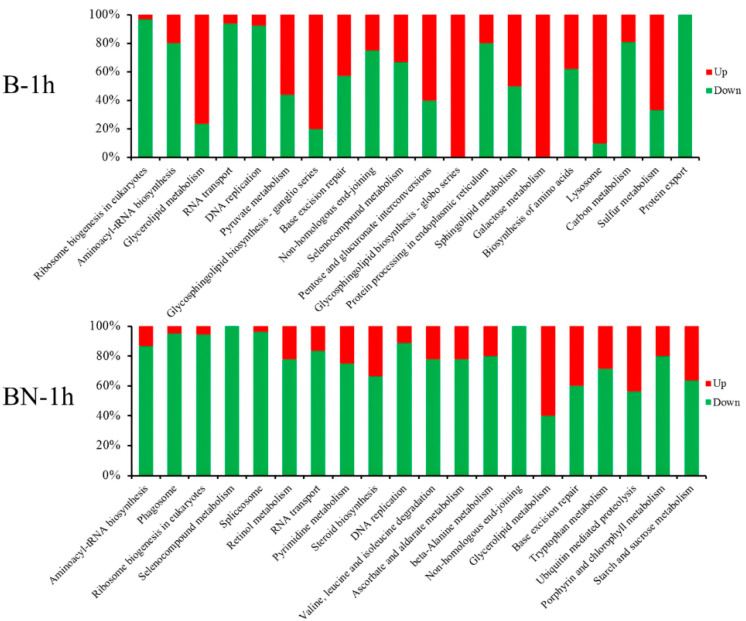
Percentage of up- or downregulated DEGs among different KEGG pathways in B-1h and BN-1h.

**Figure 6:**
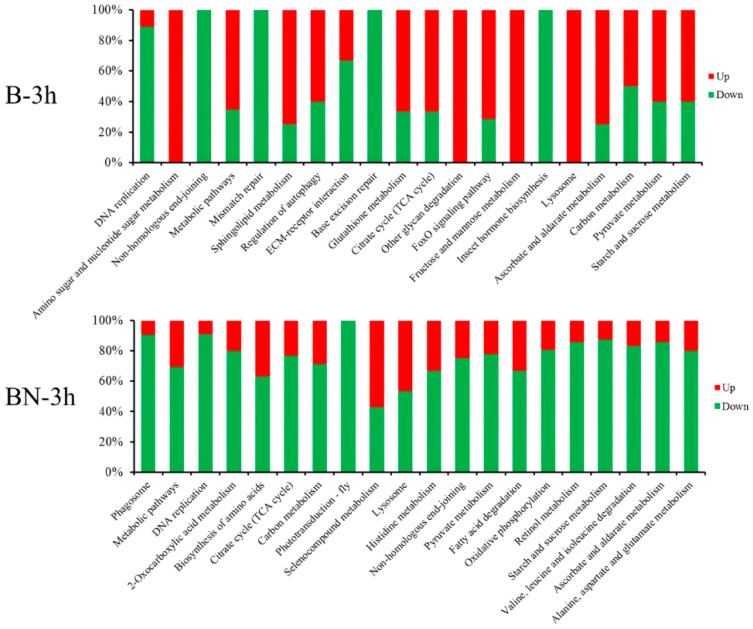
Percentage of up- or downregulated DEGs among different KEGG pathways in B-3h and BN-3h.

KEGG analyses of DEGs at different time points were performed to enrich pathways relevant to the resistance of silkworm varieties to BmNPV infection. The results showed that these DEGs were enriched in a broad range of KEGG pathways, including ribosome biogenesis, aminoacyl-tRNA biosynthesis, glycerolipid metabolism, RNA transport, DNA replication, base excision repair, selenocompound metabolism, protein processing in the endoplasmic reticulum, non-homologous end-joining, the citrate cycle (TCA cycle), pyruvate metabolism, lysosomes, and metabolic pathways. According to the *p*-values, the top 20 (or fewer than 20) KEGG pathways are listed in Figure 4 and Figure S2. Most of the DEGs involved in the top 20 (or fewer than 20) KEGG pathways were upregulated in the B variety, while a majority of DEGs involved in the top 20 KEGG pathways were downregulated in the BN variety (Figure 5, Figure 6, Figures S3 and S4). In addition, several pathways were shared in the B and BN varieties. Among the top 20 pathways, 8 pathways, namely the ribosome biogenesis in eukaryotes, aminoacyl-tRNA biosynthesis, glycerolipid metabolism, RNA transport, DNA replication, base excision repair, non-homologous end-joining, selenocompound metabolism, and 9 pathways, namely the DNA replication, non-homologous end-joining, metabolic pathways, citrate cycle (TCA cycle), lysosomes, ascorbate and aldarate metabolism, carbon metabolism, pyruvate metabolism, and starch and sucrose metabolism, were common in the B-h and BN-1h and B-3h and BN-3h groups, respectively (Figure 4A,B). Only one pathway (Metabolic pathways) was shared between the B-6h and BN-6h groups (Figure S2A,B). Interestingly, 85% of DEGs involved in the metabolic pathways in the B variety were upregulated, but about 65% of DEGs in these pathways in the BN variety were downregulated. Only two pathways, i.e., protein processing in the endoplasmic reticulum and metabolic pathways were shared between the B-9h and BN-9h groups (Figure S2C,D). Interestingly, all the DEGs involved in protein processing in the endoplasmic reticulum were downregulated in the B variety but upregulated in the BN variety.

### 3.7. Analysis of DEGs Associated with the Carbon Metabolism, DNA Replication, Starch, and Sucrose Metabolism Pathways

Enrichment analysis of DEGs identified multiple enriched pathways, including the carbon metabolism, DNA replication, and starch and sucrose metabolism pathways in two different varieties and at different time points post-infection (Figure 4 and Figure S2). The carbon metabolism pathway was enriched in the B-1h, B-3h, BN-3h, B-6h, and BN-9h groups (Figure 4 and Figure S2) but showed different change patterns. For example, most of the DEGs involved in the carbon metabolism pathway were downregulated in the B-1h and BN-3h groups (Figure 5 and Figure 6) but upregulated in the B-6h and BN-9h groups (Figures S3 and S4). Moreover, the DNA replication process was affected differently by BmNPV infection in the two varieties at 1 and 3 h time points (Figure 5 and Figure 6). About 90% of DEGs associated with the DNA replication process were downregulated in all four groups (Figure 5 and Figure 6). In addition, the starch and sucrose metabolism pathways were altered in the BN-1h, B-3h, BN-3h, and BN-6h groups (Figure 5, Figure 6, and Figure S3). The majority of DEGs involved in the starch and sucrose metabolism pathways were downregulated in the BN-1h, BN-3h, and BN-6h groups but upregulated in the B-3h group (Figure 5, Figure 6, and Figure S3).

### 3.8. Validation of DEGs by qRT-PCR

**Figure 7:**
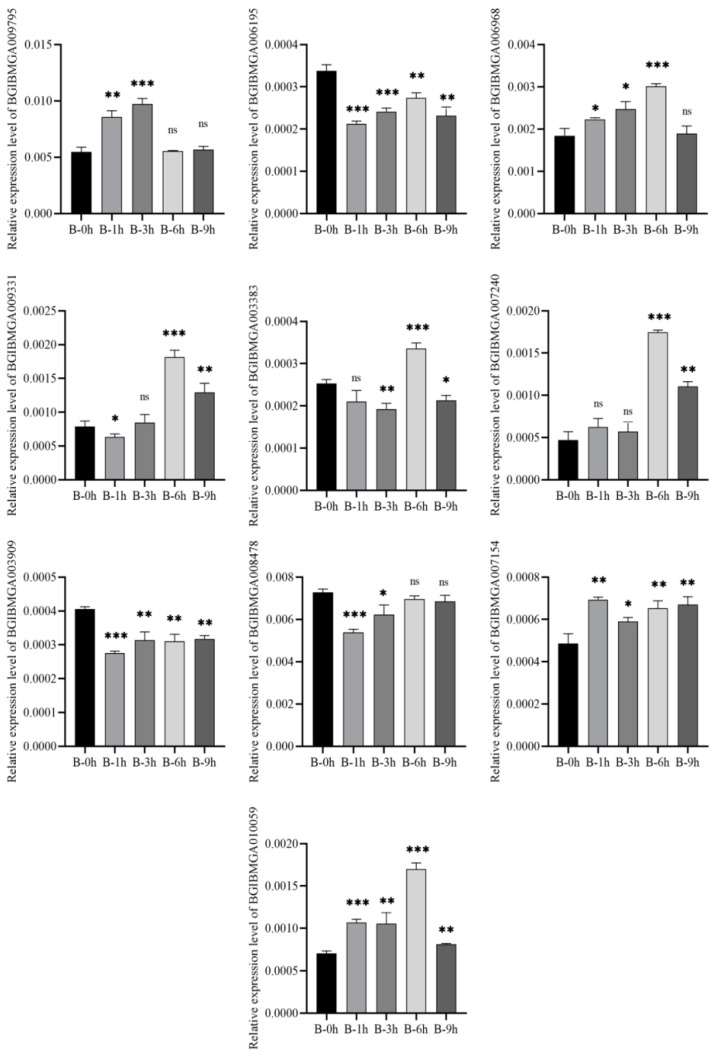
Quantification of 10 DEGs at 1, 3, 6, and 9 h after BmNPV infection in the B variety by qRT-PCR. Significant differences are indicated by asterisks (*p* < 0.05). ns, not significant. *, *p* < 0.05; **, *p* < 0.01; ***, *p* < 0.001.

**Figure 8:**
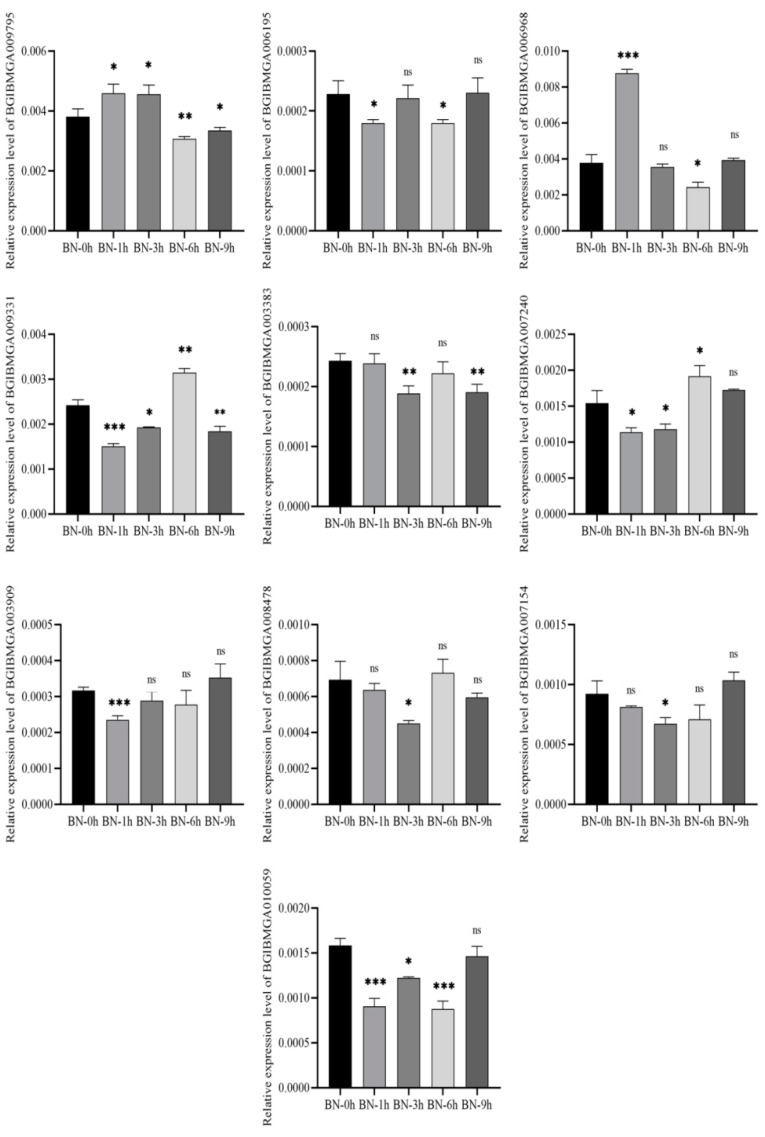
Quantification of 10 DEGs at 1, 3, 6, and 9 h after BmNPV infection in the BN variety by qRT-PCR. Significant differences are indicated by asterisks (*p* < 0.05). ns, not significant. *, *p* < 0.05; **, *p* < 0.01; ***, *p* < 0.001.

**Figure 9:**
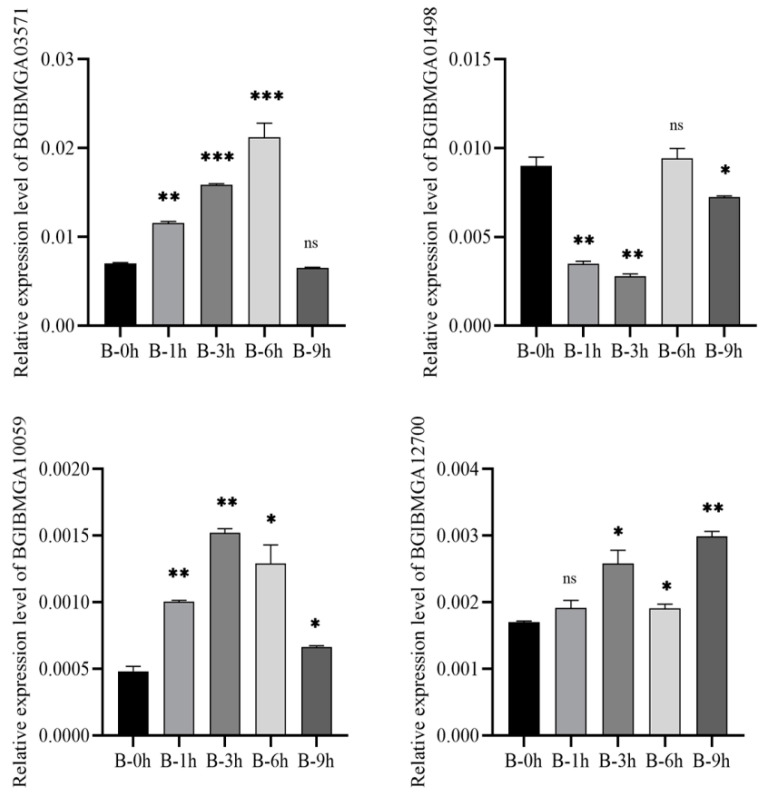
Quantification of identified four DEGs at 1, 3, 6, and 9 h after BmNPV infection in the B variety by qRT-PCR. Significant differences are indicated by asterisks (*p* < 0.05). ns, not significant. *, *p* < 0.05; **, *p* < 0.01; ***, *p* < 0.001.

**Figure 10:**
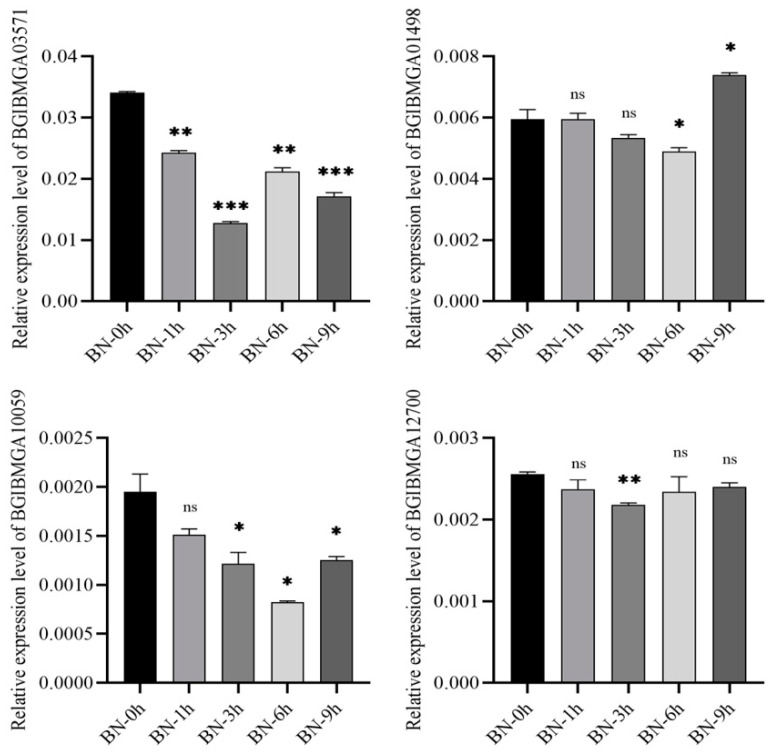
Quantification of identified four DEGs at 1, 3, 6, and 9 h after BmNPV infection in the BN variety by qRT-PCR. Significant differences are indicated by asterisks (*p* < 0.05). ns, not significant. *, *p* < 0.05; **, *p* < 0.01; ***, *p* < 0.001.

Ten DEGs in the B and BN varieties were selected for validation using qRT-PCR. The results were consistent with those from the transcriptional analyses, showing similar fold changes (Figure 7 and Figure 8). Of note, four genes (BGIBMGA03571, BGIBMGA01498, BGIBMGA10059, BGIBMGA12700) were upregulated in variety B at all time points, which was validated by qRT-PCR as well. (Figure 9 and Figure 10).

## 4. Discussion

BmNPV is one of the primary pathogens of silkworms that cause significant economic losses in the sericultural industry [26]. However, the mechanism underlying the silkworm–BmNPV interaction is still unclear, and no effective treatment is available for silkworms infected with BmNPV in sericulture. Understanding the molecular mechanism underlying the silkworm–BmNPV interaction and host defense is critical in controlling BmNPV infection in silkworms. Previous studies showed that midgut plays an important role in virus replication and host defense in silkworms [11]. Haas-Stapleton et al. demonstrated that *Spodoptera frugiperda* multiple nucleopolyhedrovirus (SfMNPV) might bind to different receptors on columnar epithelial cells of midgut, thereby promoting the initiation of its infection [27]. Investigating the interaction between BmNPV and midgut may reveal potential mechanisms underlying the resistance of silkworms to BmNPV infection. Transcriptome analysis is a powerful tool that can provide a comprehensive understanding of transcriptome changes related to specific biological processes, thus facilitating the dissection of mechanisms related to certain diseases. In this study, we carried out RNA-seq to analyze transcriptome changes in the midguts of two silkworm varieties, the B variety (susceptible) and BN (resistant), at different time points in the early stage of BmNPV infection. As a result, 2157 DEGs responding to BmNPV infection were identified by comparing infected groups with controls in the two varieties. These DEGs were potentially involved in host resistance to BmNPV infection or might be recruited by BmNPV to facilitate its infection. The two varieties showed different resistance patterns, and our transcriptome analyses at different time points revealed different transcriptional patterns as well, indicating these DEGs might explain the resistance difference in the two varieties.

As obligate intracellular parasites, viruses can only encode a limited number of genes. Therefore, they cannot replicate by themselves; they require the host’s machineries to facilitate their genome replication and virus expression, which are essential for completing their infection cycles [28,29,30]. The first 0–3 h after viral infection is critical for the infection initiation. The transcription and translation of viral genes in this period are completely dependent on the gene expression products of host cells but not the viral coding products [31]. Proteins expressed by viral genes during this period can be recognized by the host’s cytokines [32]. When the cells lack cytokines required for the immediate early gene transcription of baculovirus, although the virus can successfully enter the host cells, it cannot replicate and proliferate. Early gene expression occurs only 3–6 h after infection, and the transcription and expression of these genes depend on the products of immediate early genes of the virus [33]. The expression of these early genes provides the necessary materials for viral DNA replication. Moreover, these gene products can monitor and regulate the host cell environment to facilitate viral DNA replication. However, viral DNA replication does not affect the transcription of its early genes. The transcription of baculovirus genes during its early infection is dependent on host RNA polymerase II. Viral RNA polymerases (composed of viral proteins LEF-4, LEF-8, LEF-9, and P47), however, are mainly involved in the expression of late genes [32,33]. Thus, we hypothesized that the DEGs in the susceptible varieties would be critical for revealing the mechanism underlying silkworm resistance to BmNPV infection. From our data, seven genes, namely *Glutamate synthase* (BGIBMGA012700), *Synaptic vesicle glycoprotein 2C* (*SV2C*, BGIBMGA001498), *Calexcitin-2* (BGIBMGA008815), *Sodium-independent sulfate anion transporter isoform X1* (BGIBMGA003935), *Facilitated trehalose transporter Tret1-2 homolog* (*Tret1-2*, BGIBMGA010742), *Monocarboxylate transporter 1 isoform X2* (BGIBMGA003571), and *Transient receptor potential channel pyrexia-like* (BGIBMGA010059), were upregulated at all-time points post-infection in B variety (Figure 3A), which may be involved in virus infection or host defense. We selected four genes for qRT-PCR validation, which was consistent with the results from transcriptome analysis. Among the seven genes, overexpression of the *Tret1-X1* (BGIBMGA010742) gene in BmN cells can inhibit the proliferation and replication of BmNPV virus and effectively inhibit the expression of genes at different infection stages. The expression of *BmTret1-X1* gene can promote the expression of the BmNPV envelope protein GP64 in host cells, thus promoting the fusion of virus and host cells [34]. The other six genes have not been reported previously. Interestingly, we found that two of them, *Glutamate synthase* (BGIBMGA012700) and *Synaptic vesicle glycoprotein 2C* (*SV2C*, BGIBMGA001498), were uniquely upregulated in the variety B following BmNPV infection, indicating that they might be important host factors for BmNPV infection. More interestingly, four of them, *Calexcitin-2* (BGIBMGA008815), *Sodium-independent sulfate anion transporter isoform X1* (BGIBMGA003935), *Monocarboxylate transporter 1 isoform X2 * (BGIBMGA003571), and *Transient receptor potential channel pyrexia-like* (BGIBMGA010059), were upregulated in the B variety while downregulated in the BN variety. These four genes may be the essential host factors for virus replication, as they were upregulated in the B (susceptible) variety but downregulated in the BN (resistant) variety. Therefore, these five genes may be the key genes mediating the resistance of silkworms to BmNPV infection.

Yao et al. detected the copy numbers of virus in midguts, hemolymphs, and fat bodies at different time points post-infection by qRT-PCR. They showed that the virus can enter the midgut 2 h after oral infection or 12 h after inoculation and can complete a replication cycle in the midgut and infect hemolymphs and fat bodies in the form of budded virus (BV) within these time periods [35]. When the resistant varieties were infected with BmNPV, the expression of viral genes could be detected in the hemolymph and midgut of *B. mori* but could not be detected in the late infection stage, indicating that BmNPV can enter the resistant silkworms by blocking virus proliferation [33]. We hypothesized that the resistance of silkworms to BmNPV infection is a complex and multi-stage process. Therefore, we analyzed the transcriptome changes in the midguts of the B (susceptible) and BN (resistant) varieties at different time points in the early stage of BmNPV infection. As a result, we identified many DEGs in response to BmNPV infection, which provides an important resource for further identification of genes that are important in resistance against BmNPV infection.

When silkworms are attacked by viruses, their immune system can be activated by upregulating specific antiviral genes or downregulating specific genes that can facilitate the virus transcription and replication to resist virus infection [3,7,9,36,37]. In our study, several genes were upregulated only in the BN variety following BmNPV infection, indicating these genes are likely involved in the resistance to BmNPV infection. Interestingly, one gene, *Uncharacterized protein LOC101744317 isoform X2* (BGIBMGA004283), which was not characterized previously, was upregulated in the BN-6h group but downregulated in the B-6h group. This gene may play an important role in resistance to BmNPV, which requires further characterization.

Previous studies have shown that host cellular and humoral responses are the major strategies used by insects against microbial infection [38,39,40]. Viral infections usually cause dramatic changes in host cellular and metabolic processes [30]. A previous study suggested that oxidative phosphorylation may be the key metabolic pathway responsible for resistance to BmNPV infection in different varieties (i.e., the BN and B varieties) [41]. In this study, we showed that the pathway, i.e., protein processing in the endoplasmic reticulum, was significantly different between the B-9h and BN-9h groups (Figures S3–S6). All the DEGs involved in this pathway were upregulated in the BN-9h group but downregulated in the B-9h group (Figures S4–S6), implying that this pathway may play an important role in the resistance of silkworms to BmNPV infection. Interestingly, heat shock protein 70 (Hsp70), which was the only shared DEG in this pathway in the B and BN groups, showed opposite expression changes (Figures S5 and S6). Research in the past decade on Hsps has suggested that some members of the HSP family might directly stimulate the innate immune system in cells [42,43,44]. Furthermore, extracellular Hsps can send a strong “danger signal” to the immune system to generate a response that can help clear the invading pathogen [9,45,46]. Current evidence suggests that *Hsp70* can negatively regulate the expression of viral proteins in infected cells, and overexpression of *Hsp70* can inhibit the replication of influenza a virus in mice [47]. In this study, we found that *Hsp70* was upregulated in the BN variety, but downregulated in the B variety, indicating that *Hsp70* may contribute to resistance to BmNPV infection in the BN variety. Studies have shown that *BmHSC70-4* is continuously expressed in BmNPV-infected *B. mori*. Inhibition of *BmHSC70-4* expression can reduce BV production and delay viral DNA replication [48]. Moreover, inhibition of *Hsp90* expression can significantly reduce the titer of BmNPV, protein levels of BmNPV nucleocapsid protein 39 (VP39), and transcription level of the BmNPV gene [49]. After BmNPV infection, silkworms adopt a series of defensive strategies. Thus, studying the transcriptional changes in early-stage BmNPV infection might reveal mechanisms regarding BmNPV replication and translation, which may facilitate the development of effective methods to prevent BmNPV infection in silkworms.

To the best of our knowledge, this is the first study that explored the DEGs in different BmNPV-resistant varieties at early infection stages using transcriptome analysis. Furthermore, 6814 new transcripts were predicted (Table S5), which provides a valuable resource for further gene function study in silkworms. Characterization of host factors functioning in viral replication is critical for the development of new strategies to combat virus infection in silkworms. Our study provided insights into transcriptome regulation in midguts of silkworms in response to BmNPV infection and established a foundation for further study of the molecular mechanism underlying the resistance of silkworms to BmNPV infection.

## Figures and Tables

**Table 1:** Evaluation of resistance of the new bred silkworm varieties to BmNPV infection.

Variety Name	Mortality Rate of the 2nd Instar after BmNPV Infection with 1.0 × 10^8^ mL^−1^ Solution	Regression Equation	LC50/mL^−1^
Baiyu N	0%	Y = −7.252 + 0.510 X	2.52 × 10^9^
Baiyu	100%	Y = −10.019 + 1.603 X	2.13 × 10^5^
Qiufeng N × Baiyu N	10%	Y = −8.361 + 0.610 X	2.16 × 10^9^
Baiyu N × Qiufeng N	0%	Y = −8.233 + 0.702 X	1.99 × 10^9^
Qiufeng × Baiyu	100%	Y = −10.240 + 1.692 X	1.13 × 10^5^
Baiyu × Qiufeng	100%	Y = −12.080 + 1.904 X	2.21 × 10^5^

**Table 2:** Summary of RNA-seq datasets.

Sample	Raw Reads	Raw Bases	Clean Reads	Clean Bases	Valid Ratio (Base)	Q30 (%)	GC Content (%)	Ratio of Reads Mapped to Genome
B-0h-1	28562592	3570324000	27765390	3467734429	97.12%	95.25%	47.00%	79.89%
B-0h-2	28876928	3609616000	28096334	3509155838	97.21%	95.34%	46.00%	81.33%
B-0h-3	28794488	3599311000	27820182	3474441859	96.53%	94.78%	45.00%	80.55%
B-1h-1	28088422	3511052750	27287032	3407989919	97.06%	95.20%	46.50%	80.47%
B-1h-2	28960774	3620096750	28142744	3514894097	97.09%	95.26%	46.00%	80.98%
B-1h-3	28012158	3501519750	27141482	3389744667	96.80%	95.03%	46.50%	81.02%
B-3h-1	28319422	3539927750	27575244	3444110971	97.29%	95.46%	47.00%	80.58%
B-3h-2	28880206	3610025750	28152056	3516148247	97.39%	95.49%	45.50%	79.56%
B-3h-3	28080402	3510050250	27354780	3416555313	97.33%	95.44%	46.00%	80.66%
B-6h-1	28368774	3546096750	27573602	3443838179	97.11%	95.26%	46.50%	81.40%
B-6h-2	28045628	3505703500	27025310	3375100921	96.27%	94.60%	46.00%	79.09%
B-6h-3	28399900	3549987500	27571816	3443565824	97.00%	95.17%	46.00%	80.72%
B-9h-1	28317000	3539625000	27475118	3431461235	96.94%	95.13%	46.50%	79.94%
B-9h-2	28119310	3514913750	27307226	3410564463	97.03%	95.24%	45.50%	80.96%
B-9h-3	28648046	3581005750	27760426	3467084956	96.81%	95.04%	46.00%	81.28%
BN-0h-1	28666864	3583358000	27841768	3477331144	97.04%	95.17%	47.50%	81.01%
BN-0h-2	28298442	3537305250	27464334	3430189736	96.97%	95.16%	47.00%	80.85%
BN-0h-3	28012628	3501578500	27274002	3406499188	97.28%	95.42%	47.00%	81.28%
BN-1h-1	28205818	3525727250	27406492	3422936978	97.08%	95.21%	46.50%	79.69%
BN-1h-2	28279168	3534896000	27416632	3424133438	96.86%	95.02%	47.50%	78.98%
BN-1h-3	28512350	3564043750	27675112	3456531187	96.98%	95.20%	46.50%	80.65%
BN-3h-1	28011992	3501499000	27204406	3397702496	97.03%	95.23%	46.50%	78.92%
BN-3h-2	28291120	3536390000	27537810	3439446648	97.25%	95.45%	47.00%	81.19%
BN-3h-3	28764132	3595516500	27990216	3495896532	97.22%	95.35%	48.00%	80.88%
BN-6h-1	28219152	3527394000	27113062	3385619587	95.98%	94.55%	46.50%	77.41%
BN-6h-2	28662614	3582826750	27623744	3449506275	96.27%	94.82%	47.00%	80.39%
BN-6h-3	28650550	3581318750	27580236	3444099370	96.16%	94.79%	46.00%	81.16%
BN-9h-1	28010608	3501326000	27051472	3378073060	96.47%	94.95%	46.00%	80.31%
BN-9h-2	28861830	3607728750	27704832	3459568321	95.89%	94.56%	46.50%	80.07%
BN-9h-3	28443426	3555428250	27444454	3427214063	96.39%	94.89%	47.50%	80.19%

## Data Availability

Raw sequencing reads of all samples reported in this study have been deposited into the NCBI Sequence Read Archive from SRR20959739-SRR20959768 under Bioproject PRJNA867118.

## Supplementary Materials

The following supporting information can be downloaded at: https://www.mdpi.com/article/10.3390/v14112405/s1, Figure S1: Percentage of up- or downregulated DEGs in different groups; Figure S2: KEGG pathway classification analysis of the DEGs; Figure S3: Percentage of up- or downregulated DEGs among different KEGG pathway in 6 h; Figure S4: Percentage of up- or downregulated DEGs among different KEGG pathway in 9 h; Figure S5: Effects of BmNPV infection on “protein processes in the endoplasmic reticulum” in B-9h; Figure S6: Effects of BmNPV infection on “protein processes in the endoplasmic reticulum” in BN-9h; Table S1: Designed primers of 14 DEGs; Table S2: All the DEGs identified in this study; Table S3: Number of DEGs at different groups after BmNPV infection; Table S4: The DEGs identified in different groups; Table S5: New transcripts predicted in this study.
