# Supplementary material for: Identification of Key Genes Involved in Resistance to Early Stage of BmNPV Infection in Silkworms
Source: Viruses. 2022 Oct 29;14(11):2405. doi: 10.3390/v14112405 (PMC9694779; doi:10.3390/v14112405)
Supplement: Supplementary file 1 [file viruses-14-02405-s001.zip › Figures S1¿CS6.pdf]

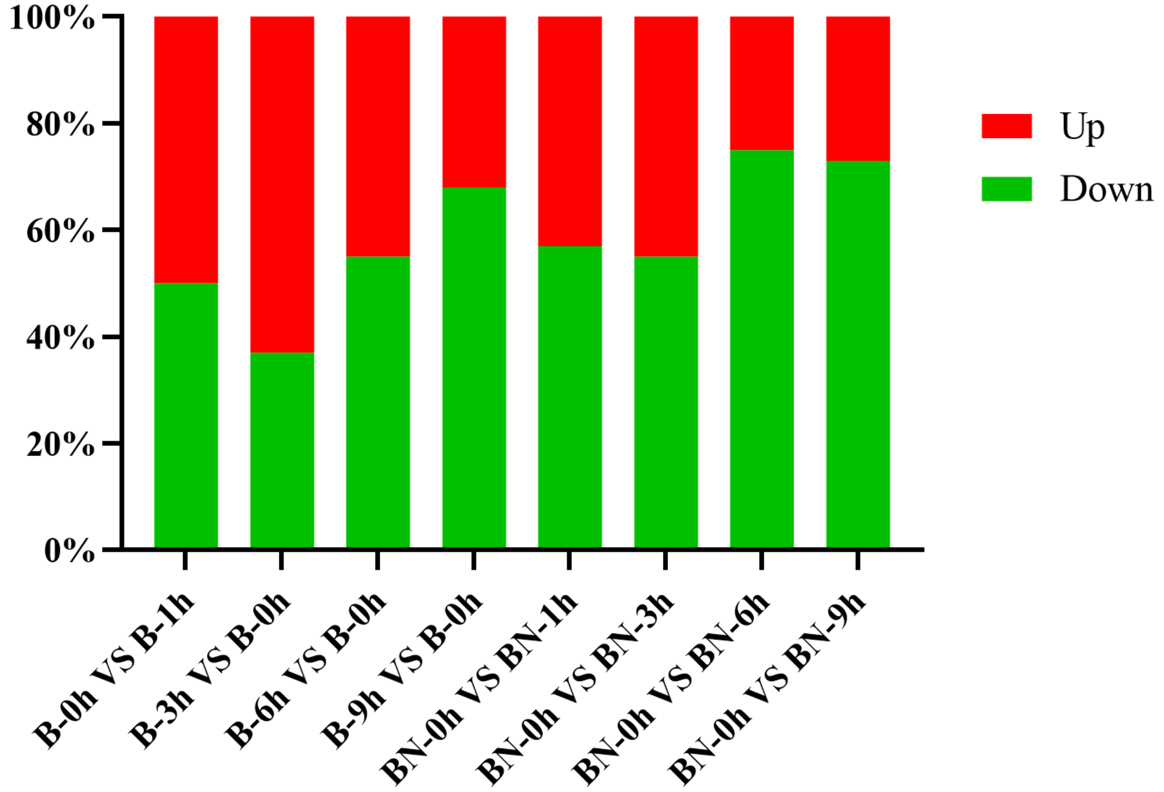

Figure S1. Percentage of up- or downregulated DEGs in different groups.

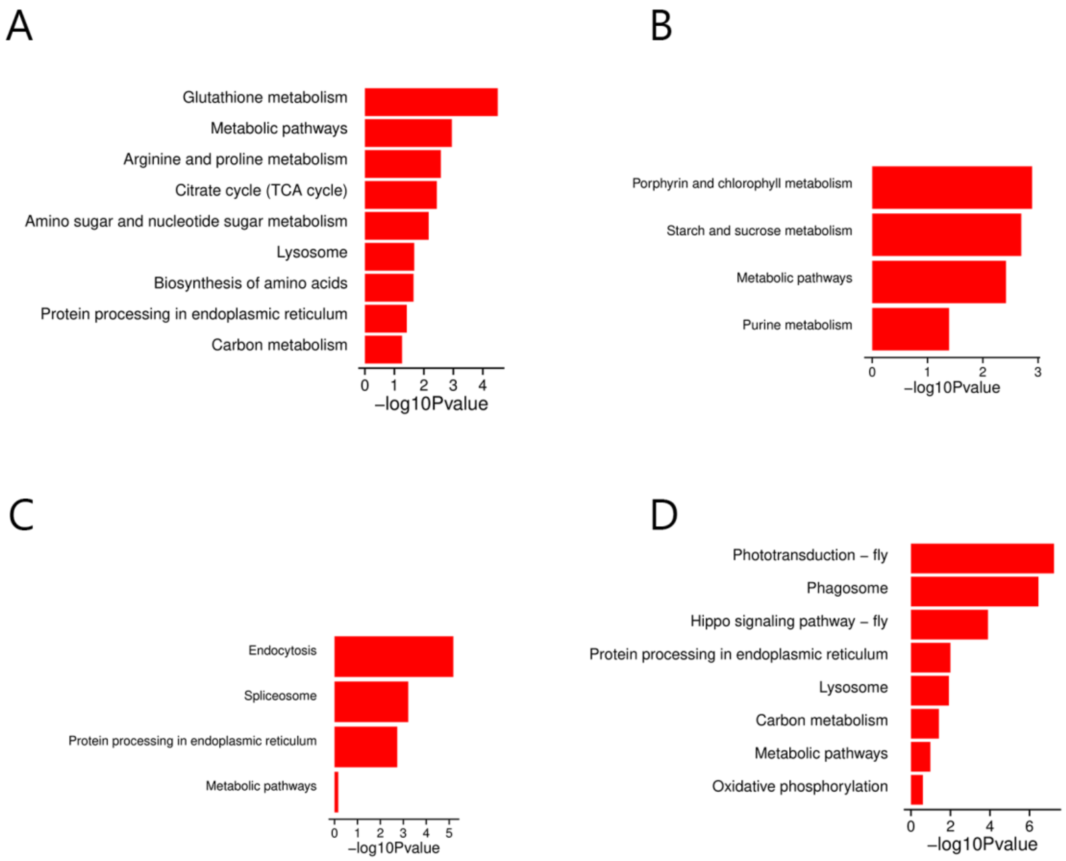

Figure S2. KEGG pathway classification analysis of the DEGs.

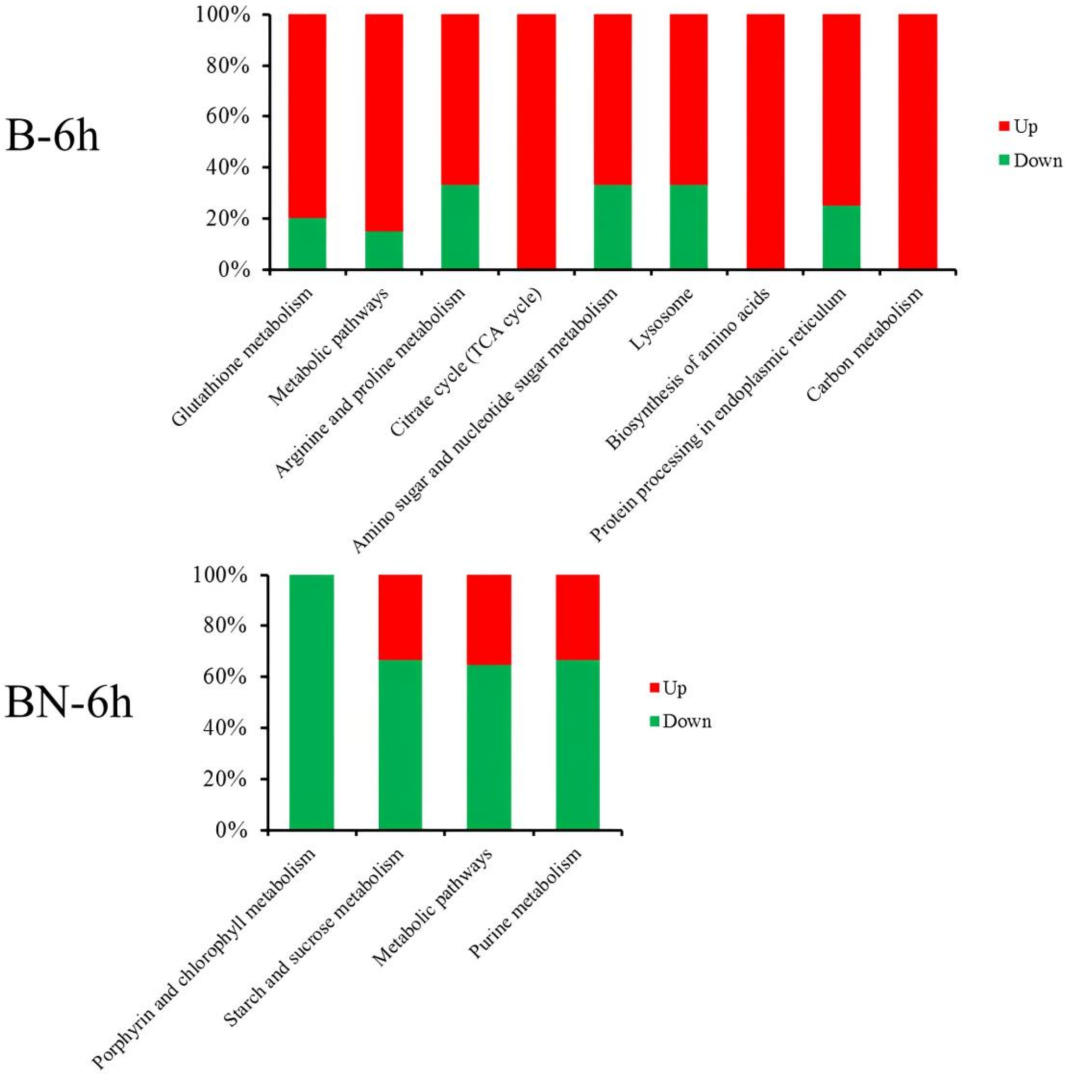

Figure S3. Percentage of up- or downregulated DEGs among different KEGG pathway in 6h.

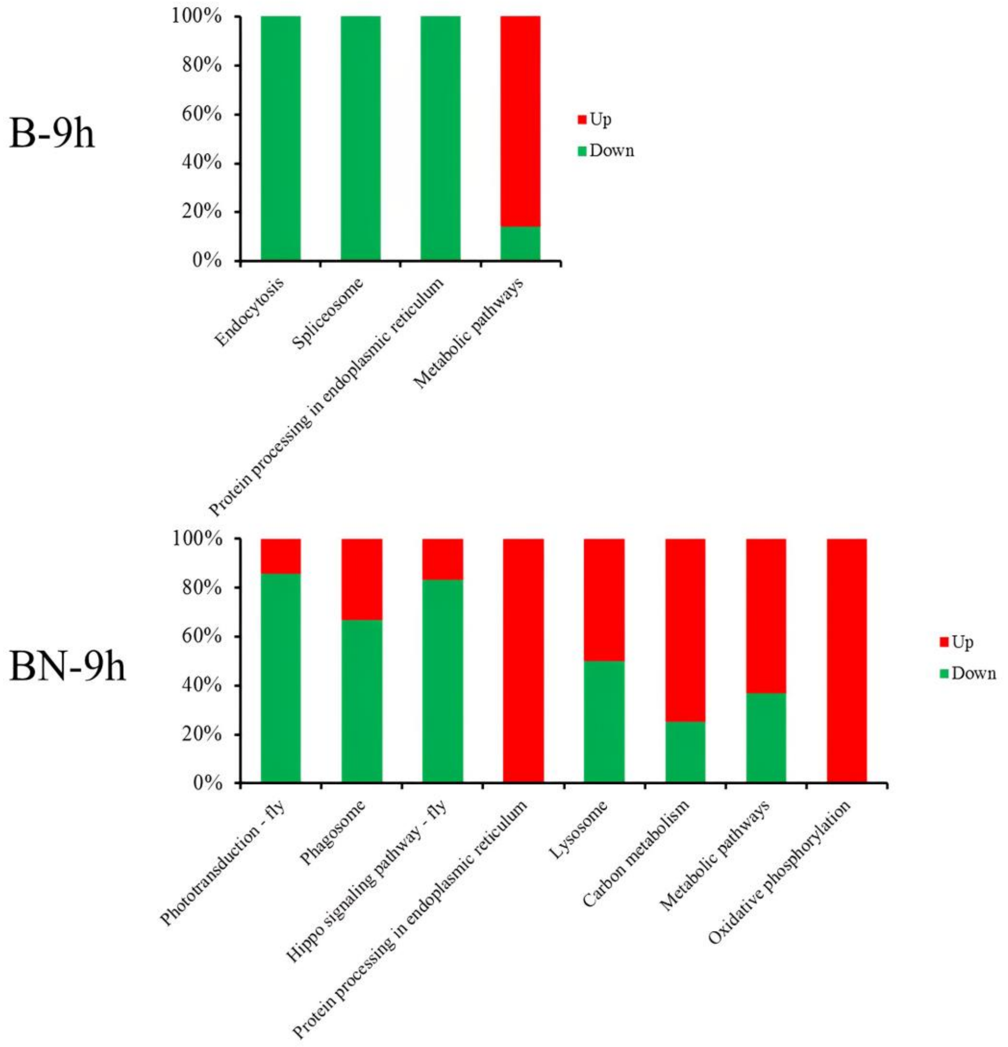

Figure S4. Percentage of up- or downregulated DEGs among different KEGG pathway in 9h.

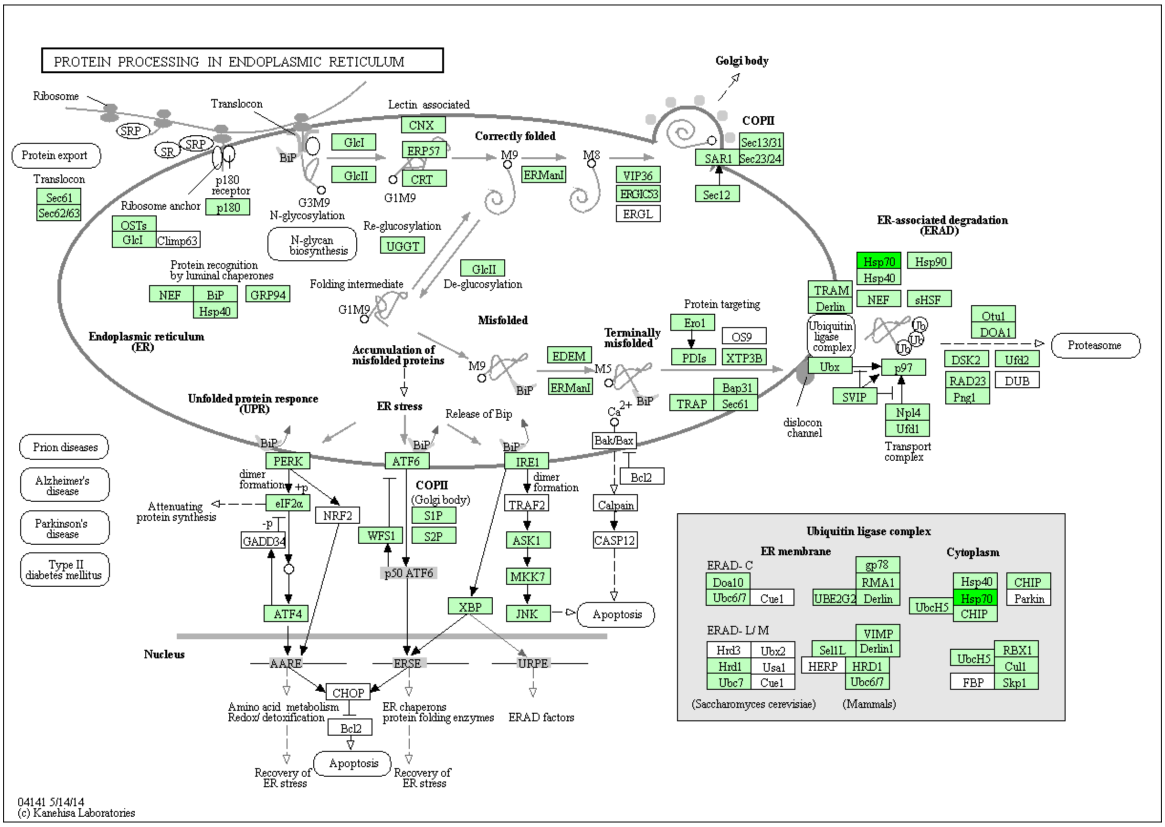

Figure S5. Effects of BmNPV infection on "protein processes in the endoplasmic reticulum" in B-9h.

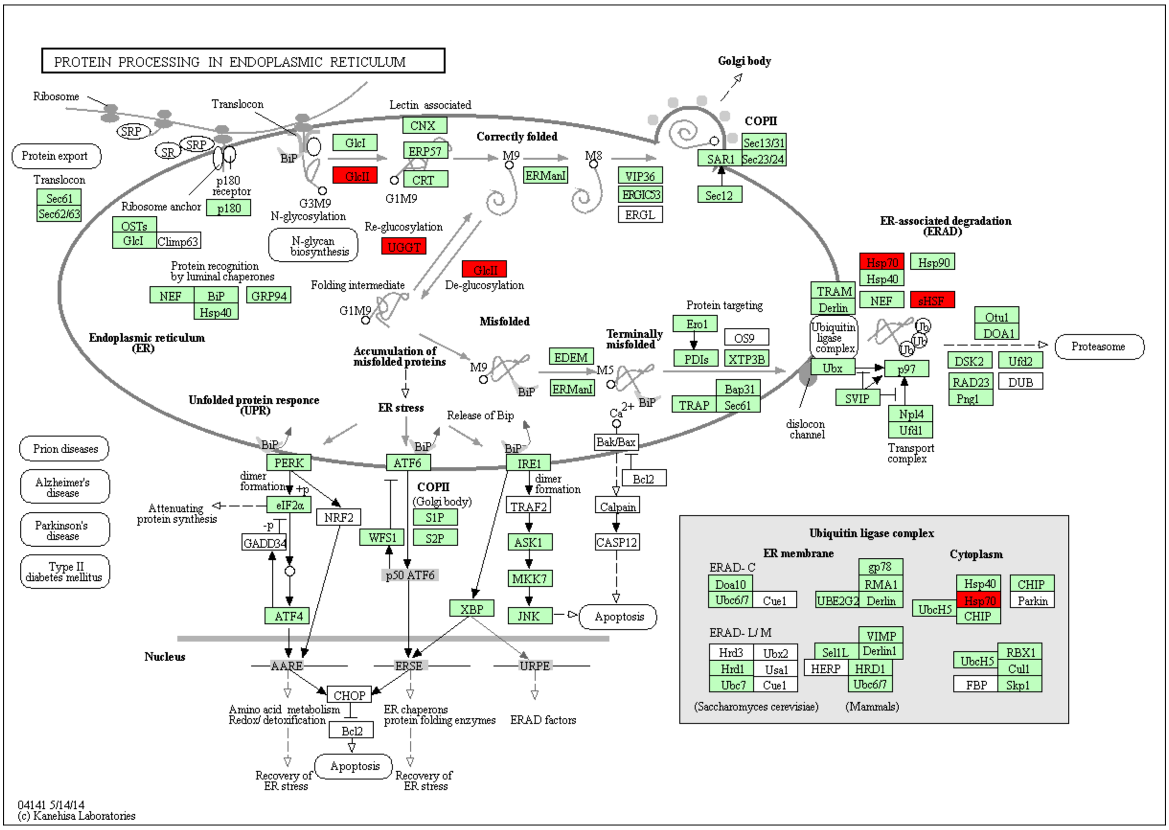

Figure S6. Effects of BmNPV infection on "protein processes in the endoplasmic reticulum" in BN-9h.
