# Supplementary material for: Identification of Key Genes Involved in Resistance to Early Stage of BmNPV Infection in Silkworms
Source: Viruses. 2022 Oct 29;14(11):2405. doi: 10.3390/v14112405 (PMC9694779; doi:10.3390/v14112405)
Supplement: Supplementary file 1 [file viruses-14-02405-s001.zip › Table S1.pdf]

Table S1 Designed primers of 14 DEGs

Gene	Primer
Actin-3	F: 5′- CGGCTACTCGTTCACTACC -3′<br>R: 5′- CCGTCGGGAAGTTCGTAAG -3′
Q-*BGIBMGA003383*	F: 5′- TACCAAGGCTTCTGATGATT -3′<br>R: 5′- ACTGCGTTCGTCTTCTATT -3′
Q-*BGIBMGA008478*	F: 5′- GCTGTTAATCCTGTAGATACC -3′<br>R: 5′- GTGTGCCTTCCACTGTAA -3′
Q-*BGIBMGA003909*	F: 5′- CTTGGTATGACTACACTGAGA -3′<br>R: 5′- TCGTGAACCTGTTGTAAGTA -3′
Q-*BGIBMGA006195*	F: 5′- CACTTCGCACCATATACCA -3′<br>R: 5′- CTCAATTACTCTCCGTCCTATA -3′
Q-*BGIBMGA009331*	F: 5′- CTCACGACTCGGTGTTCA -3′<br>R: 5′- CTTCATCATCACTGCCATCA -3′
Q-*BGIBMGA009795*	F: 5′- CAGGTGAACATCGTGACA -3′<br>R: 5′- ATCTTCCGTTGCTCGTTAA -3′
Q-*BGIBMGA007240*	F: 5′- GGAACTACACCTGACAGAAT -3′<br>R: 5′- CCACGACATTGCCACTAT -3′
Q-*BGIBMGA006968*	F: 5′- GTAACACGACCATCCTATCT -3′<br>R: 5′- ACCTTATCCTGCCATTGAC -3′
Q-*BGIBMGA007154*	F: 5′- ATACAACGGATTCGGAGTC -3′<br>R: 5′- GAACAATGAACCACACTGAG -3′
Q-*BGIBMGA010059*	F: 5′- ATGTCAGCCGCAATCAAT -3′<br>R: 5′- CACGAACCACTCCATCAA -3′
Q-*BGIBMGA003571*	F: 5′- GCGTTCATTCTTGTTGGCGAAT -3′<br>R: 5′- ACATAGAATGCCACGGTGTAGC -3′
Q-*BGIBMGA001498*	F: 5′- CGTCAGCACTTCCGAGGAGATG -3′<br>R: 5′- CCAAGCGAGTCTGCCACTGAAC -3′
Q-*BGIBMGA010059*	F: 5′- AGCAGCAGCATTCGATCCAGAG -3′<br>R: 5′- CATGTTCGGTGCATCGTCAGC -3′
Q-*BGIBMGA012700*	F: 5′- CGAAGCCTGCGGTGTTGGTT -3′<br>R: 5′- TTGATGAGGGATAGCGGTCAGT -3′
