# Supplementary material for: Identification of Key Genes Involved in Resistance to Early Stage of BmNPV Infection in Silkworms
Source: Viruses. 2022 Oct 29;14(11):2405. doi: 10.3390/v14112405 (PMC9694779; doi:10.3390/v14112405)
Supplement: Supplementary file 1 [file viruses-14-02405-s001.zip › Table S3.pdf]

Table S3 Number of DEGs at different groups after BmNPV infection

	Total	Up	Down
B-1h VS B-0h	1247	655（52.5%）	592（47.5%）
B-3h VS B-0h	424	271（63.9%）	153（36.1%）
B-6h VS B-0h	150	67（44.7%）	83（55.3%）
B-9h VS B-0h	90	30（33.3%）	60（66.7%）
BN-1h VS BN-0h	910	454（50%）	456（50%）
BN-3h VS BN-0h	456	238（52.2%）	218（47.8%）
BN-6h VS BN-0h	112	32（28.6%）	80（71.4%）
BN-9h VS BN-0h	99	30（30.3%）	69（69.7%）
